# Whole-Genome Sequence and Classification of 11 Endophytic Bacteria from Poison Ivy (*Toxicodendron radicans*)

**DOI:** 10.1128/genomeA.01319-15

**Published:** 2015-11-19

**Authors:** Phuong N. Tran, Nicholas E. H. Tan, Yin Peng Lee, Han Ming Gan, Steven J. Polter, Lucas K. Dailey, André O. Hudson, Michael A. Savka

**Affiliations:** a Monash University Malaysia Genomics Facility, Monash University Malaysia, Jalan Lagoon Selatan, Bandar Sunway, Petaling Jaya, Selangor, Malaysia; b School of Science, Monash University Malaysia, Jalan Lagoon Selatan, Bandar Sunway, Petaling Jaya, Selangor, Malaysia; c Thomas H. Gosnell School of Life Sciences, Rochester Institute of Technology, Rochester, New York, USA

## Abstract

Here, we report the whole-genome sequences and annotation of 11 endophytic bacteria from poison ivy (*Toxicodendron radicans*) vine tissue. Five bacteria belong to the genus *Pseudomonas*, and six single members from other genera were found present in interior vine tissue of poison ivy.

## GENOME ANNOUNCEMENT

*Toxicodendron radicans*, or poison ivy (PI), is a plant that produces an oily allergen called urushiol (1, 2). Urushiol is an oleoresin within the sap of the plant and is thought to regulate the retention of water. PI is a member of the *Anacardiaceae* family and is commonly found throughout central and eastern North America and in the Canadian Maritime. The PI plant is well known for causing urushiol-induced contact dermatitis. Besides causing an immune response that leads to dermatitis (3), microorganisms harbored by the plant may be involved in causing secondary lesions in persons that come in contact with the plant (4). We are not aware of any studies that describe the identification of bacteria that associate with PI. As such, we embarked on a project to isolate and identify bacterial endophytes from the internal vine tissue of PI. Eleven species of bacteria were sequenced, five of which are different species of *Pseudomonas*, of which two have complete taxonomic designations, *Pseudomonas libanensis* and *Staphylococcus hominis.*

Vine tissue of PI plants was collected in early May (early growing season) in Rochester, NY. Vines were surface sterilized, and the internal stem tissue was prepared axenically, inoculated in tryptic soy broth medium, and cultured for 5 days, followed by plating and incubation. Morphologically distinct colonies were subcultured, and genomic DNA isolation was performed using the E.Z.N.A. bacterial DNA kit (Omega Bio-Tek, Norcross, GA) and prepared for sequencing using the Nextera XT kit (Illumina, San Diego, CA), according to the manufacturer’s instructions. Sequencing was performed on the MiSeq sequencer (Illumina, San Diego, CA) located at the Monash University Malaysia Genomics Facility. Raw FASTQ reads for each library were adapter trimmed using Trimmomatic (version 0.33) and subsequently error corrected and *de novo* assembled into contigs with the SPAdes genome assembler (version 3.5.0) (5). To obtain a preliminary genus/species identity, the 16S rRNA from each assembly was extracted by RNAmmer (6) and searched against the NCBI 16S database using BLASTN (7). Genus-level identity was further augmented by a similarity search against other common housekeeping gene segments, such as *rpoB*, *gyrB*, and *recA*. Species-level identification was also attempted using JSpecies (8). The key annotation properties for the 11 genomes and taxonomy information are presented in Table 1.

**TABLE 1.** Sequencing, annotation, and classification of the 11 bacterial endophytes isolated from internal vine tissues of the poison ivy (*Toxicodendron radicans*) plant

GenBank accession no.	Organism	Genome size (bp)	No. of contigs	*N*_50_ (bp)	Coverage (×)	Top hit based on 16S rRNA (as of Sept 2015)	Nucleotide identity (%)^a^				
							16S rRNA	*rpoB*	*recA*	*gyrB*	ANI
LGIU00000000	*Arthrobacter* sp. RIT-PI-e	3,459,090	104	69,834	145	*Arthrobacter agilis* CCM 2390	98.86	–	88.26	–	–
LHOX00000000	*Enterococcus* sp. RIT-PI-f	3,221,568	25	235,705	112	*Enterococcus gallinarum* LMG 13129	99.81	89.45	82.52	78.80	–
LHOZ00000000	*Frigoribacterium* sp. RIT-PI-h	3,445,346	134	48,954	84	*Frigoribacterium* sp. MEB024	100	98.79	96.00	97.00	93.72
LGIT00000000	*Klebsiella* sp. RIT-PI-d	4,256,263	20	2,264,346	117	*Klebsiella oxytoca* strain KCTC 1686	98.62	92.98	87.59	86.68	77.63
LGIS00000000	*Pantoea* sp. RIT-PI-b	5,271,693	35	377,981	104	*Pantoea rodasii* strain LMG 26273	98.51	92.78	–	90.31	–
LHOY00000000	*P. libanensis* RIT-PI-g	6,164,468	54	256,980	77	*P. libanensis* CIP105460	100	98.19	–	97.24	–
LGIR00000000	*Pseudomonas* sp. RIT-PI-a	4,732,857	35	320,153	106	*Pseudomonas rhizosphaerae* strain ih5	99.34	97.50	94.68	91.88	90.24
LHPA00000000	*Pseudomonas* sp. RIT-PI-o	6,103,656	54	304,793	63	*Pseudomonas mandelii* strain DSM 17967	98.23	94.68	94.01	88.09	–
LHPC00000000	*Pseudomonas* sp. RIT-PI-q	7,446,015	125	132,429	52	*Pseudomonas frederiksbergensis* strain CIP 106887	97.9	95.61	–	94.90	–
LIGE00000000	*Pseudomonas* sp. RIT-PI-r	6,518,981	49	318,071	65	*Pseudomonas fluorescens* strain CECT 378	99	92.45	91.72	89.75	–
LHPB00000000	*S. hominis* RIT-PI-k	5,602,085	103	254,527	66	*S. hominis* strain ATCC 27844	100	98.84	97.13	100	97.20

a ANI, average nucleotide identity calculated by JSpecies. Dashes indicate that the gene for comparison was not sequenced in the corresponding type strain.

The presence of a variety of *Pseudomonas* species isolated in this study bears many similarities to endophytic populations found in other plants, such as grapevines (9). In addition, the prevalence of endophytic *Pseudomonas* is known to occur in grapevine flowers (10) and Norway spruce seeds (11). The prevalence of *Pseudomonas* may suggest a mutualistic symbiotic relationship with plants, in which the bacteria promote plant growth and development and, in turn, the plant provides an environment to promote bacterial colonization, usually through sugar production via photosynthesis (12). The ability of these bacterial strains to metabolize urushiol oil produced by the PI plant warrants further investigation.

### Nucleotide sequence accession numbers.

The nucleotide sequences have been deposited at DDBJ/EMBL/GenBank under the accession numbers provided in Table 1. 
